# A 10-Year Retrospective Case Series on Wound Dehiscence Following Corneal Transplant

**DOI:** 10.7759/cureus.25184

**Published:** 2022-05-21

**Authors:** Kit May Chow, Rohanah Alias

**Affiliations:** 1 Department of Ophthalmology, Hospital Kuala Lumpur, Kuala Lumpur, MYS

**Keywords:** dalk, pk, trauma, deep anterior lamellar keratoplasty, penetrating keratoplasty, corneal transplant outcome, post corneal transplant, wound dehiscence

## Abstract

Background and objective

Corneal transplants are associated with multiple well-known complications, one of them being wound dehiscence. It results in unfavorable outcomes, such as ocular structure damage, graft failure, repeated surgeries, and possibly a poor prognosis in terms of vision quality. Although the wound site may appear well-healed clinically, as the strength at the graft-host junction is weak, the site is susceptible to dehiscence. Wound dehiscence can be secondary to suture removal and/or trauma. In this study, we assessed the incidence of wound dehiscence secondary to trauma following corneal transplant and evaluated its occurrence in terms of patient age, type of corneal transplant performed, duration between corneal transplantation and wound dehiscence, mechanism of injury, and final outcome.

Methods

This was a case series that included all patients who suffered from wound dehiscence secondary to trauma post-corneal transplant between January 1, 2009, and December 31, 2019, at Hospital Kuala Lumpur.

Results

A total of 492 patients underwent corneal transplant surgery during the 10-year study period. Based on specified inclusion and exclusion criteria, only 13 patients were eligible for inclusion in this study. The incidence of wound dehiscence secondary to trauma post-penetrating keratoplasty (PK) was low (2.64%). Twelve patients had undergone PK, while one patient had undergone deep anterior lamellar keratoplasty (DALK). Blunt ocular trauma post-transplant can cause wound dehiscence regardless of patient age and duration post-corneal transplantation. Males are at a higher risk as their active lifestyle contributes to higher exposure to ocular injury.

Conclusion

As corneal transplant patients are at life-long risk of wound dehiscence post-transplant, they must be counseled about this possible risk and the need to take adequate precautions in their daily lives. Based on our findings, the use of newer technologies and partial- rather than full-thickness corneal transplants should be explored further.

## Introduction

Corneal transplants have rapidly evolved over the past 10 years. Among the different types of corneal transplant surgeries, penetrating keratoplasty (PK) is the procedure with the longest history. PK is the most common type of corneal transplant procedure in which a full-thickness cornea is replaced. Subsequently, partial-thickness corneal transplants, such as deep anterior lamellar keratoplasty (DALK) and Descemet stripping endothelial anterior lamellar keratoplasty (DSAEK), have been developed. In DALK and DSAEK, only the diseased layers of the cornea are replaced, thereby reducing the risk of intraoperative suprachoroidal hemorrhage and improving postoperative wound stability [1].

Corneal transplants have multiple well-known complications, such as graft rejection, graft failure, recurrence of the primary disease, infection, glaucoma, cataracts, and wound dehiscence. The incidence of wound dehiscence post-corneal transplant is low, ranging from 0.6% to 5.7%. When wound dehiscence occurs, it results in unfavorable outcomes, such as ocular structure damage, graft failure, repeated surgeries, and possibly a poor prognosis in terms of vision quality [2]. Although the wound site may appear well-healed clinically, the site can be susceptible to dehiscence since the strength at the graft-host junction is weak [3]. Wound dehiscence can also occur secondary to suture removal and/or trauma.

In this study, our goal was to analyze the incidence of wound dehiscence secondary to trauma after corneal transplant and evaluate its occurrence in terms of patient age, type of corneal transplant performed, duration between corneal transplantation and wound dehiscence, mechanism of injury, and final outcome.

## Materials and methods

Study design

This was a retrospective case series.

Inclusion and Exclusion Criteria

All patients who had wound dehiscence secondary to trauma post-corneal transplant from January 1, 2009, to December 31, 2019, at Hospital Kuala Lumpur were included. The exclusion criteria were as follows: wound dehiscence not secondary to trauma, cases outside the specified study period, and corneal transplant surgeries not performed at Hospital Kuala Lumpur.

Ethical Approval

Prior to data collection, we obtained ethical approval from the Medical Research and Ethics Committee, Ministry of Health, Malaysia (NMRR-21-1120-59629). The study adhered to the tenets of the Declaration of Helsinki for human research.

Data Collection

The universal sampling method was used for all cases that fulfilled the inclusion and exclusion criteria. Corneal transplant patients were identified via a search of the patient database of Hospital Kuala Lumpur. Relevant data extracted from the patients’ medical records included patient age and gender, type of surgery performed (PK or DALK), indication for the transplant, the total number of transplants prior to trauma, the time interval between the transplant and wound dehiscence, mechanism of injury, and graft survival post-injury.

The type of corneal transplant performed was recorded, as different methods are used to replace different layers of the cornea. The types of corneal transplants included PK, a full-thickness corneal transplant, and DALK, a partial-thickness corneal transplant where the cornea is replaced only up to the stromal layer, keeping the recipient’s own Descemet’s membrane and endothelium in situ. The indications for a corneal transplant reflected the fact that Hospital Kuala Lumpur is a tertiary center with a number of ophthalmology subspecialties. Hence, corneal transplant cases included a wide range of age groups and diseases.

The total number of transplants performed prior to injury was included to show that wound dehiscence can occur irrespective of the number of transplants performed. The mechanism of injury was included to identify the most common causes of injuries, thereby enabling prevention measures to be recommended [1]. The final graft outcome was included to shed light on the potential impact of traumatic injury on future vision quality.

Statistical analysis

Results were summarized into frequencies, represented as simple percentages. No inferential statistics were performed.

## Results

The search of the database identified 492 patients who had undergone corneal transplant surgery in the 10-year period from January 1, 2009, to December 31, 2019. Based on the inclusion and exclusion criteria, only 13 patients were eligible for inclusion in this study.

Table 1 provides a summary of the data collected.

**Table 1:** Summary of patients who sustained wound dehiscence post-corneal transplant *First PK performed at age 42 years for corneal decompensation with secondary glaucoma and uveitis secondary to a bee sting. **First PK performed aged 60 years for fungal keratitis. ***First PK performed aged 60 years for bullous keratopathy with traumatic dislocation of the lens. ****First PK performed aged 10 years for congenital glaucoma with bullous keratopathy. Second PK performed aged 12 years for primary graft failure. Third PK performed aged 18 years for graft failure PK: penetrating keratoplasty; DALK: deep anterior lamellar keratoplasty

No.	Age, years	Sex	Type of corneal transplant	Indication for transplant	No. of transplants	Time (post-transplant to injury)	Mechanism of injury	Final graft outcome (survived/failure)
1	2.7	M	PK	Congenital glaucoma complicated by descemetocele and microperforation	1	21 days	Excessive rubbing	Failure
2	3	F	PK	Perforated corneal ulcer secondary to exposure keratopathy with underlying Stickler syndrome with glaucoma	1	2 years 8 months	Struck in the eye while playing	Failure (phthisis bulbi)
3	20	M	PK	Advanced keratoconus	1	1 month 7 days	Got punched in the eye during a quarrel	Survived
4	0.3	M	PK	Ectatic cornea secondary to corneal staphyloma with anterior segment dysgenesis	1	3 days	Finger poking of the eye	Failure
5	67	M	PK	Fuchs endothelial dystrophy with secondary glaucoma	1	5 years	Elbow in the eye	Survived
6	62	F	PK	Avellino corneal dystrophy	1	3 years	Struck in the eye by a fruit	Survived
7	2.6	F	PK	Congenital glaucoma with corneal ulcer	1	1 day	Struck bed railing while playing	Failure (phthisis bulbi)
8	52	M	PK	Failed graft	2*	8 months	Finger poking of the eye while face-washing	Survived
9	61	F	PK	Failed graft	2**	11 months	Struck in the eye	Survived
10	26	M	PK	Advanced keratoconus	1	6 years	Got punched in the eye during a quarrel	Survived
11	28	M	DALK	Keratoconus	1	8 years	Struck in the eye	Survived
12	78	M	PK	Failed graft	2***	3 years	Struck the edge of the bed	Failure
13	28	M	PK	Failed graft	4****	4 years	Slipped and fell face down	Survived

The age of the patients included in the study ranged from 0.3 years to 78 years, with a mean of 33.1 years. Nine out of the 13 patients (69.2%) were males; 12 patients with wound dehiscence underwent PK and only one patient with wound dehiscence had DALK performed. Most of the patients with wound dehiscence (9/13, 69.2%) had undergone only one corneal transplant prior to ocular trauma.

The duration from surgery to wound dehiscence varied from one day to eight years. All of them had blunt ocular trauma. However, only seven out of 13 (53.8%) of the corneal grafts survived. Almost half (46.2%) of the patients’ grafts failed, with two of them leading to phthisis bulbi.

## Discussion

The incidence of wound dehiscence secondary to trauma post-PK at Hospital Kuala Lumpur between January 1, 2009, and December 31, 2019, was 2.64%, which was lower than the incidence found in other studies (0.6-5.7%) [2]. The duration from surgery to wound dehiscence varied from one day to eight years, pointing to continued wound site weakness post-transplant and susceptibility to dehiscence. In terms of the interval between PK and trauma, Foroutan et al. reported a mean interval of 15.6 months [4], while Jafarinasab et al. reported a mean interval of 44.1 months [5], and Singar et al. reported a range of 0.03-34 months [6]. These findings suggest that wound dehiscence can occur any time postoperatively due to the weakness of the graft-host junction. According to Pettinelli et al., it takes two to three years for human eyes to achieve two-thirds of their normal corneal tensile strength post-corneal transplant, and the original tensile strength is never regained in a transplanted cornea [7].

In this study, nine of the 13 (69.2%) patients with wound dehiscence were males; this could be attributed to the fact that males are more physically active and this contributes to higher exposure to ocular injury. A study by Rehany et al. also found that males had a higher risk of traumatic wound dehiscence [8].

Studies conducted in various countries have reported that wound dehiscence post-PK occurs most commonly in younger age groups. In these studies, the most common indication for PK was keratoconus [5-10]. In our study, the ages of the patients with wound dehiscence ranged from 0.3 to 78 years, with a mean of 33.1 years. Four of the patients were in the pediatric age group, and four were young adults in their 20s. The other five patients were aged more than 50 years.

In contrast to previous reports, six of the 13 (46.2%) patients in our study had underlying glaucoma, and PK was performed due to the complications of glaucoma. Of the four corneal transplant patients in the pediatric age group, three had underlying congenital glaucoma, and one had anterior segment dysgenesis. In one patient, wound dehiscence occurred due to excessive eye rubbing. In the other three patients, wound dehiscence was due to an injury sustained while playing, with these patients sustaining more severe injuries and their eyes later becoming phthisis bulbi. In a study by McClellan et al. on PK in children, none of the grafts in the congenital group failed due to wound dehiscence [9]. However, in a study by Patel et al. on pediatric corneal transplantation, trauma was the third most common cause of graft failure [10].

In the present study, three of the four patients in their 20s had a corneal transplant due to keratoconus, whereas the other patient had underlying congenital glaucoma and had undergone four PK procedures prior to the injury. The cause of wound dehiscence in two of these patients was a punch in the eye during a quarrel, whereas the causes in the other two cases were accidental ocular injuries. In all four cases, the entire replacement cornea remained clear at the most recent follow-up visits. According to the Australian Corneal Graft Registry, graft failure secondary to eye injury accounts for 8% of all graft failures in keratoconus [11].

In this study, the indications for a corneal transplant among patients older than 50 years old varied. However, each three of the five patients had undergone two PK procedures prior to the injury. All five patients sustained wound dehiscence secondary to accidental injuries. According to a multivariate analysis by Weisbrod et al., graft survival in repeat PK was poorer compared to that in primary PK [12]. The two- and five-year graft survival rates for repeat PK in their study were 63.9% and 45.6% respectively, whereas those of primary grafts were 78.8% and 64.6% respectively [12].

As corneal transplant patients carry a life-long risk of developing wound dehiscence post-transplant, they must be properly counseled about this possible risk [13]. In addition, patients must be constantly reminded during follow-up visits about the need to be careful during activities of daily living and to wear protective eyewear [14]. Young active patients, particularly males, should be reminded of the need to avoid physical confrontations due to the potential for trauma and wound dehiscence. Very young children are also a high-risk group for wound dehiscence, as they are unable to protect themselves from injury. In the event of injuries, adequate pain relief must be given to ensure patient comfort and avoid wound dehiscence secondary to forceful Valsalva during crying. By nature, children enjoy playing and learn from playing. However, post-corneal transplant, children should wear an eye shield at all times while playing. In addition, play should be limited to safer, low-intensity activities.

Of note, of the 13 patients who had wound dehiscence during the 10-year period, only one patient had undergone DALK. This suggests that new technologies and partial- rather than full-thickness corneal transplants should be explored for this procedure. Replacing only the damaged part of the cornea rather than the whole cornea better preserves corneal structure and integrity [15-16].

A limitation of this study pertains to the rarity of wound dehiscence secondary to trauma, despite a search of records for the past 10 years. A well-designed prospective study is needed to compare the incidence of wound dehiscence secondary to trauma post-PK versus post-DALK.

## Conclusions

Corneal transplant patients are at life-long risk of developing wound dehiscence post-transplant. Hence, they must be appropriately counseled about this possible risk and about the need to take adequate precautions in their daily lives. In this retrospective study, only one of the 13 patients who experienced wound dehiscence underwent DALK, with the rest undergoing PK. This finding suggests that newer technologies and partial- rather than full-thickness corneal transplants should be explored going forward.
